# Using the WHO Surgical Safety Checklist to Direct Perioperative Quality Improvement at a Surgical Hospital in Cambodia: The Importance of Objective Confirmation of Process Completion

**DOI:** 10.1007/s00268-017-4198-x

**Published:** 2017-10-16

**Authors:** Naomi Y. Garland, Sokhavatey Kheng, Michael De Leon, Hourt Eap, Jared A. Forrester, Janice Hay, Palritha Oum, Socheat Sam Ath, Simon Stock, Samprathna Yem, Gerlinda Lucas, Thomas G. Weiser

**Affiliations:** 1 Department of Surgery, Section of Acute Care Surgery, Stanford University, 300 Pasteur Drive, Stanford, CA 94305 USA; 2 World Mate Emergency Hospital, P.O. Box 339, National Road 5, Romcheck IV, Rattanaka, Battambang, Cambodia; 3 Department of Surgery, St. Elizabeth’s Medical Center, CMP 2, Room 2041, 736 Cambridge St, Brighton, MA 02135 USA

## Abstract

**Background:**

The WHO surgical safety checklist (SSC) is known to prevent postoperative complications; however, strategies for effective implementation are unclear. In addition to cultural and organizational barriers faced by high-income countries, resource-constrained settings face scarcity of durable and consumable goods. We used the SSC to better understand barriers to improvement at a trauma hospital in Battambang, Cambodia.

**Methods:**

We introduced the SSC and trained data collectors to observe surgical staff performing the checklist. Members of the research team observed cases and data collection. After 3 months, we modified the data collection tool to focus on infection prevention and elicit more accurate responses.

**Results:**

Over 16 months we recorded data on 695 operations (304 cases using the first tool and 391 cases with the modified tool). The first tool identified five items as being in high compliance, which were then excluded from further assessment. Two items—instrument sterility confirmation and sponge counting—were identified as being misinterpreted by the data collectors’ tool. These items were reworded to capture objective assessment of task completion. Confirmation of instrument sterility was initially never performed but rectified to >95% compliance; sponge counting and prophylactic antibiotic administration were consistently underperformed.

**Conclusions:**

Staff complied with communication elements of the SSC and quickly adopted process improvements. The wording of our data collection tool affected interpretation of compliance with standards. Material resources are not the primary barrier to checklist implementation in this setting, and future work should focus on clarification of protocols and objective confirmation of tasks.

## Introduction

Surgical services and capacity are increasingly recognized as an integral component of essential healthcare [1]. Assuring high-quality surgical and perioperative care requires organizational capacity that is frequently weak in resource poor settings. Organizational focus on surgical quality is often not prioritized, and addressing infrastructure barriers to compliance with basic standards of care is difficult. In many countries, regulatory oversight is inadequate [2]. There is little published regarding viable solutions to achieving basic standards of surgical care in international settings.

In 2008 the World Health Organization (WHO) developed the surgical safety checklist (SSC), a perioperative communication and safety tool to improve compliance with basic standards [3]. The WHO SSC represents an internationally accepted set of tasks performed at critical perioperative moments. Some items are reminders to communicate specific information; others confirm presence or functionality of operating theater equipment, or basic infection prevention measures such as prophylactic antibiotic administration.

Given its international acceptance as a standard for improving quality perioperative care, we used objective measures of checklist performance to assess compliance with perioperative standards at an orthopedic trauma hospital in Battambang, Cambodia. We aimed to use our assessment of compliance, and subsequent identification of problems or gaps in perioperative care, to improve adherence to the infection prevention components of the WHO SSC.

## Methods

### Setting

World Mate Emergency Hospital (WMEH) is an 89-bed trauma hospital in Battambang, Cambodia, which predominantly manages orthopedic trauma. The hospital was originally established by an Italian non-governmental organization (NGO) to treat landmine victims. Over time, it has evolved to address the growing number of injuries from road traffic accidents and occupational injuries throughout the western portion of the country. WMEH has a catchment population of 1.2 million and performs approximately 2000 cases per year. The surgeons are predominantly Cambodian, with the exception of one British expatriate general surgeon [4].

The WMEH management team developed an interest in quality improvement and perioperative care, inviting our research team to implement and assess the use of the WHO SSC. In July 2015, the checklist was introduced during a 1-week interactive workshop followed by a week of implementation and coaching led by one of the authors (TGW). Staff were guided through a process of checklist modification to meet their specific institutional needs, participated in role playing exercises, and practiced implementing the checklist with supervision and guidance in the operating theater (OT).

### Data collection

We focused on identifying the performance of specific checklist items, including both communication elements and perioperative tasks (Table 1). Data collectors were trained to document specific item performance via a combination of observation and verbal confirmation with the surgical team. A member of the research team observed data collection in the operating theater during approximately 20% of the cases. Cases were observed on a convenience basis, while the nurses assigned to collecting data were on duty. Data collectors were not empowered to intervene if they noted that an item was not performed based on local operating theater practice and culture. Perioperative data were initially collected via a paper form, which was subsequently converted to a mobile REDCap tool for ease of data acquisition and storage, though the content of the survey was not altered.

**Table 1:** Questions comprising data collection tools 1 and 2

Paper and electronic tool 1 questions
Did team confirm patient identity, site, and procedure? (Y/N)
Did surgeon state how long case would take? (Y/N)
Did surgeon estimate blood loss? (Y/N)
Did anesthetist confirm equipment check? (Y/N)
Did scrub nurse confirm implants and equipment are available? (Y/N)
Did team members introduce themselves? (Y/N)
Antibiotics given? (Y/N)
If yes, time of antibiotic infusion: (time)
Is imaging available? (Y/N/Not applicable)
Was instrument sterility confirmed? (Y/N)
Time of incision: (time)
Were sponge and instrument counts performed? (Y/N)

Modified tool 2 questions
Were antibiotics given in the OT? (Y/N)
(If “no”) Were antibiotics given before the patient went to the OT? (Y/N)
Time of antibiotics and incision:
Name of antibiotic:
Was a sterile marker inside the instrument tray? (Y/N)
Did the sterile marker or tape inside the tray change color? (Y/N)
Was a gauze count performed at the beginning of the case? (Y/N)
How many sponges were counted?
Did the surgeon enter the room with wet hands prior to donning sterile gown and gloves? (Y/N)
What was available for hand washing at the sinks outside the OT? (Medicated soap and water/Soap and water/Gel hand sanitizer/Water only/Other:__)
Were new surgical gloves used? (Y/N)
Were there tears or damage in any of the gowns? (Y/N)
Were the gowns replaced? (Y/N)
What was the patient’s skin washed with? (Iodine/Chlorhexidine/Alcohol wash/Soap and water/Other/No prep)
Were there tears or damage in any of the drapes? (Y/N)
Were the drapes replaced? (Y/N)
Was a gauze count performed at the end of the case? (Y/N)
How many gauze swabs were counted?

Data collection included demographic information, presenting diagnosis, name of surgical procedure, nature of injury and admission (traumatic or urgent), time of incision and antibiotic administration, compliance with specific checklist items, and compliance with infection prevention measures. Initially, we collected data regarding the surgical wound classification but found this was not logistically feasible at this facility. A copy of data collection tool 1 and tool 2 can be found in Appendices 1 and 2, respectively.

### Modification of data collection tool

**Fig. 1:**
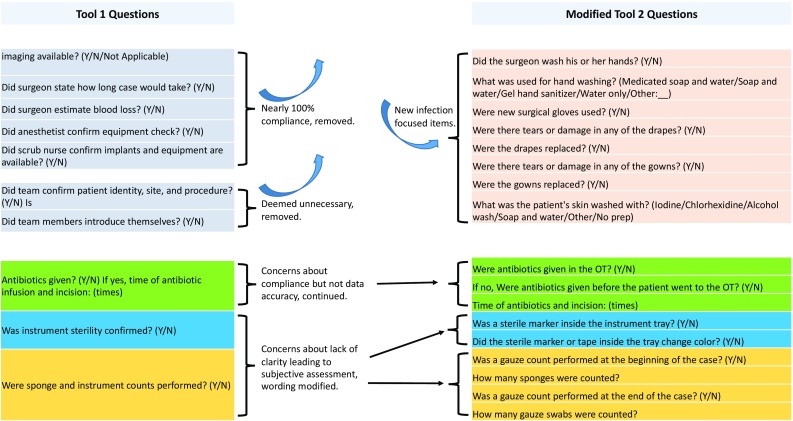
The original data collection tool, tool 1, was modified by removing those items with near 100% compliance or found to be contextually unnecessary, rewording unclear questions with a focus on objectivity, and adding more infection prevention focused items

After 24 weeks of data collection the results were presented to the surgical team. Modifications addressed three issues: (1) discrepancies between objective completion of checklist items and reporting by the surgical team and data collectors, (2) an increased focus on infection prevention measures, and (3) removal of high-compliance items (Fig. 1).

After examining the data collected with the first tool, the research team noted significant discrepancies between recorded and objective task completion in the OT for two items: confirmation of instrument sterility and post-procedure sponge counting. We modified the wording of those questions to focus the data collector on objective observations rather than verbal confirmation or interpretation by the surgical team. For confirmation of sterility, the data collectors were asked to verify that they had visualized an indicator in the tray and that it had changed color under sterilizing conditions.

The second tool was also modified to incorporate more elements specific to infection prevention, adding in items to assess use of sterile surgical gloves, hand washing and availability of soap, presence and quality of sterile gowns and drapes, and skin preparation.

Finally, items with which the team was already in high compliance at the start of checklist implementation were removed to decrease survey fatigue. The data collectors were retrained to use the updated tool 2, with an emphasis on objective verification rather than subjective assessment (Table 1). Data were collected using the updated tool for an additional 30 weeks.

### Clean cases for analysis of antibiotic administration

Analysis for cases of antibiotic administration within 60 min was limited to clean cases and a small selection of clean-contaminated cases, as defined by standard CDC definitions [5]. This included any procedures with hardware implants, delayed primary closures, tendon repairs, skin grafts, and hernia repairs with mesh. Excluded cases for analysis of timely antibiotic administration included debridements, hardware removals, placement of external fixation, closed reductions with K wires, and craniotomies. Some clean cases typically done without preoperative antibiotics were also excluded, including hernia repairs without mesh, breast biopsies, and thyroidectomies.

## Results

**Fig. 2:**
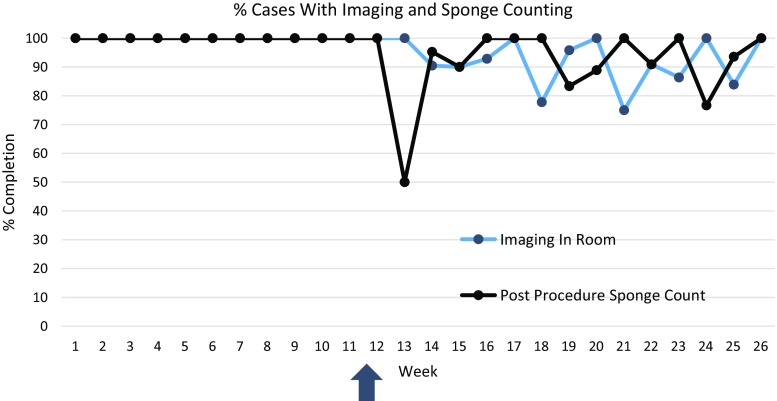
Percent of cases with documentation of imaging available in room and post-procedure sponge counting. During weeks 1–11, a paper tool was used, and at week 12 (*arrow*), an electronic tool was introduced

We directly observed 695 surgical cases over 16 months; 304 using the first tool and 391 cases with the second, modified tool (Table 1). Early data showed some items being consistently completed during >99% of cases, most of which were related to communication or reliant upon durable and non-durable supplies. One item, antibiotic administration within 60 min, was identified as being performed inconsistently, and lastly a third category of items was identified for which reporting was inconsistent with what members of the research team observed during data collection. This last group included post-procedure sponge counting and confirmation of instrument sterility. After converting to an electronic version and emphasizing confirmation of objective observation of compliance rather than subjective assessment, we noted more variability in responses (Fig. 2). After introduction of the modified tool 2, documented compliance was more consistent with what the research team was observing.

**Fig. 3:**
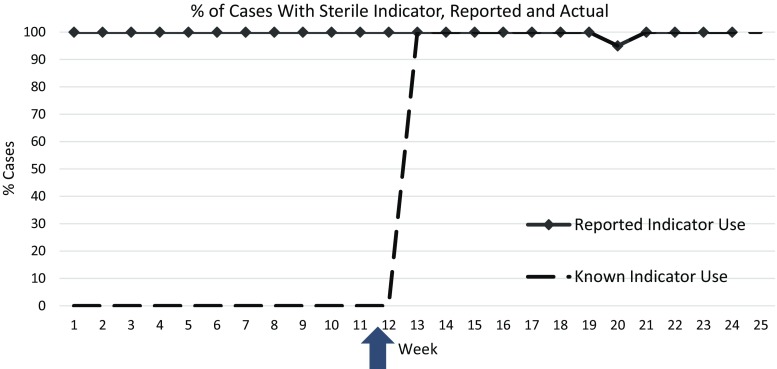
Percent of cases with documented confirmation of instrument sterility. During weeks 1–11, a paper tool was used, and at week 12 (*arrow*), an electronic tool was introduced, and a surrogate sterile indicator was developed

Measured compliance with checklist items fell into three broad categories of adherence: consistent compliance with no intervention, poor compliance improved with intervention, and consistently poor compliance (Table 2). Items consistently completed were those requiring team communication, anesthesia and surgical equipment verification, skin decontamination, estimation of blood loss, and case duration. One item, confirmation of instrument sterility, had initial poor compliance with subsequent improvement. Initially, there were no chemical sterile indicators available, although the item was documented as compliant for 100% of cases (Fig. 3). At week 12, sterile processing staff began placing chemical sterile indicator tape inside the tray. Subsequently, recording of sterile indicator presence and color change from appropriate sterilization conditions resulted in compliance in greater than 95% of cases.

**Table 2:** Percent of cases in which individual checklist items were completed

	N (%)
**Completed with great consistency**	
*Communication elements*	
Surgeon stated length of case	304/304 (100%)
Surgeon estimated blood loss	304/304 (100%)
*Durable resources*	
Anesthetist confirmed equipment check	304/304 (100%)
Anesthetist confirmed patient monitoring	304/304 (100%)
Scrub nurse confirmed implants and equipment	302/304 (99.3%)
*Behavior*	
Surgeon entered room with wet hands in sterile fashion	390/391 (99.7%)
*Process and consumable resources*	
Appropriate soap available at sink outside OT	390/391 (99.7%)
New surgical gloves used	304/304 (100%)
Sterile marker or tape inside the instrument tray changed color	390/391 (99.7%)
Surgical drapes free of tears or damage, or replaced if damaged	384/391 (98.2%)
Surgical gowns free of tears or damage, or replaced if damaged	380/391 (97.1%)
Patient’s skin prepped with iodine solution	391/391 (100%)
Imaging available, if necessary	277/304 (91.1%)
**Improved with intervention**	
*Process and consumable resource*	
Marker of sterility present in surgical instrument tray	639/695 (91.9%)
**Inconsistently performed**	
*Process and non-durable resource*	
Antibiotics given within 60 min of skin incision for “clean” case	271/402 (67.4%)
*Process*	
Surgical sponge count performed at start of case (data from tool 2 only)	328/391 (83.9%)
Surgical sponge count performed at end of case (data from tool 2 only)	84/391 (21.5%)

**Fig. 4:**
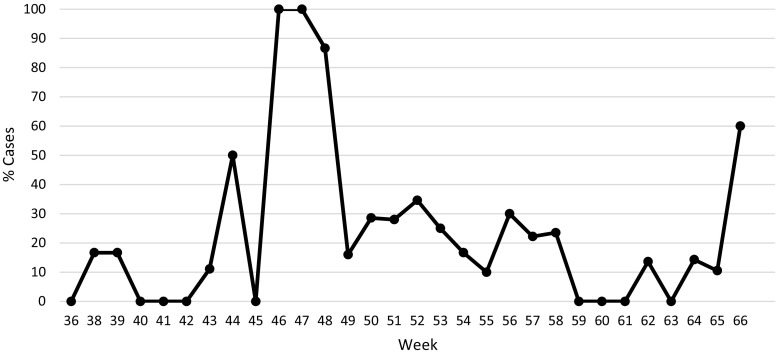
Percent of cases with post-procedure sponge count as assessed using the second tool, which required input of number of sponges counted if data collector indicated a count had been performed

Two items had consistently poor compliance: surgical sponge counting and antibiotic administration within 60 min prior to skin incision. Pre-procedure sponge counting was completed with 83.9% compliance, with post-procedure counting completed less frequently (21.5%) (Fig. 4). Informal interviews with the OT staff revealed that post-procedure sponge counting was limited to operations in which the incision was subjectively determined to be large enough that a sponge could be erroneously left behind. Operations with small incisions, for example operations on distal extremities, did not receive post-procedure sponge counts.

**Fig. 5:**
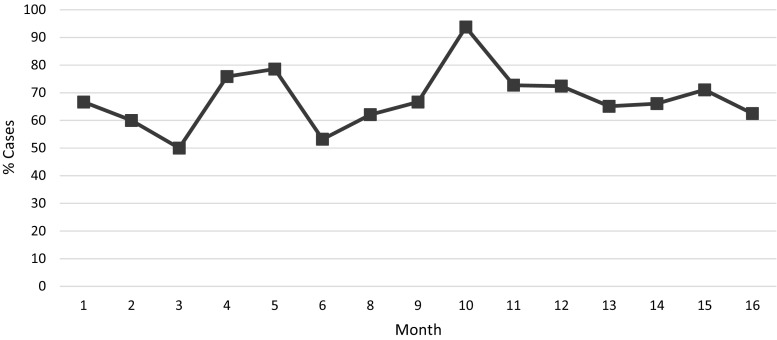
Percent of clean cases with prophylactic antibiotics administered within 60 min of skin incision

Of the 402 “clean” surgical cases observed, appropriate timing of prophylactic antibiotic administration within 60 min of skin incision was achieved in 67.4% of operations (Fig. 5). Antibiotics were administered by the anesthesia team in the OT. The antibiotic data were presented to the surgical team, though no resource or process deficiency prohibiting appropriate antibiotic administration was identified.

## Discussion

Our pilot site in Cambodia, WMEH, has the material resources necessary to implement the WHO surgical safety checklist. The staff readily engaged in the communication elements of the checklist, easily adopting new behaviors introduced through checklist implementation. After identification of readily correctable deficiencies such as lack of sterility indicators in surgical trays, the staff were able to quickly adopt specific improvements implemented by hospital management through a defined protocol.

Our experience with data collection demonstrated that there were inconsistencies between subjective perception that a checklist item has been completed and objective verification that the task had been performed. This was seen in confirmation of instrument sterility, peri-procedure sponge counting, and correct surgical site confirmation, suggesting that effective checklist implementation and assurance of perioperative quality of care demand a detailed understanding of the process of care, and not subjective interpretation of compliance. For example, for the question “was a post procedure sponge count performed?” the initial interpretation was whether or not a sponge had been left in the wound. This interpretation led to incongruent affirmative responses, when the count was not performed. Likewise, the question regarding confirmation of instrument sterility was interpreted as asking if the instruments were presumed to be sterile, not if there was objective, visual confirmation of sterility. These seemingly minute question interpretation differences can lead to challenges in accurate data collection and true quality assessment, and highlight the need for data collection tool validation and modification.

Checklist compliance variability arose almost exclusively in processes involving subjective clinical decision making. The timely administration of antibiotics, sponge counting, and site marking were all done inconsistently. Informative for future hospital improvement work, these processes were performed at the subjective discretion of the surgical team. Prophylactic antibiotics administration within 60 min prior to skin incision is recommended for “clean” or “clean-contaminated” cases, which often relies on the surgeon’s clinical assessment. While the clinical determination can be straightforward for some cases, such as orthopedic hardware implants, it can be more subjective for others, such as skin closures and wound management procedures.

These results indicate a need for further work in understanding, categorizing, and improving decision making with these complex perioperative processes and emphasize the importance of objective verification over subjective assessment. Notably, these potential areas for improvement do not require additional material resources, as is often the assumed need in public health issues. While resource constrained, the steady NGO financial support to WMEH provides the resources necessary for providing basic, quality perioperative care.

There are limitations to the study. First, given the perioperative observation by research members, the Hawthorne effect may have had an influence, particularly regarding communication elements such as estimating blood loss and case duration or behavior-based elements like surgical sponge counting. Second, ambiguity in the clinical determination of wound class could have led to inaccuracy in reporting on proper timing of prophylactic antibiotics. For example, all cases documented as “delayed primary closures” were considered to require antibiotics. However, often a small, superficial wound being closed in addition to other “dirty” procedures such as debridement were labeled as a delayed primary closure. Thus, it is possible that compliance with timely antibiotic administration is higher than the data indicate.

Third, the case observation schedule was reliant upon constrained nursing scheduling, and may have introduced a selection bias. The employed data collectors worked during the day and were instructed to preferentially observe “clean” cases if multiple cases were occurring. It is likely this potential bias is small as research team members did observe night cases over one week and observed primarily debridements and closed reductions, cases that do not require strict infection prevention measures. Furthermore, the surgical staff all perform both day and night shifts, so no staff work exclusively during the day or night, potentially biasing checklist habits.

Two questions, operative site marking and team introduction, were removed from the data analysis because of more fundamental issues; site marking was unknown to the surgical team and required separate education and training; thus, we felt it did not fit into an appropriate pattern for data analysis. Team introduction was deemed unnecessary early on in checklist introduction because the surgical team at WMEH is relatively small, and members knew each other well.

Coupling of the perioperative checklist adherence data to patient outcomes measurements has emerged as a focus for future work. There were attempts to record rates of surgical site infections at WMEH using indirect methods of patient chart review. However, data was found to be unreliable due to charting inconsistencies. Ongoing work includes the development and implementation of a robust and accurate infection surveillance program.

This work was conceived as a means to better understand how perioperative safety and infection prevention improvements can be achieved in low-resource settings. We found that lack of material resources was not a significant barrier to quality care at this pilot hospital in Cambodia; rather, checklist items that were open to subjective clinical decision making were the most difficult for surgical teams to perform consistently. Key lessons learned involved the importance of ongoing data collector training and contextually specific data collection tool improvement. Notably, institution-specific protocols and policies are needed to enhance consistency in perioperative tasks involving clinical decision making. Based on the pattern of checklist completion at WMEH, we emphasize the need for an in-depth evaluation of these complex perioperative processes in low-resource settings for improved surgical patient safety.
